# Diagnostic uncertainty and urinary tract infection in the emergency department: a cohort study from a UK hospital

**DOI:** 10.1186/s12873-020-00333-y

**Published:** 2020-05-19

**Authors:** Laura J. Shallcross, Patrick Rockenschaub, David McNulty, Nick Freemantle, Andrew Hayward, Martin J. Gill

**Affiliations:** 1 Institute of Health Informatics, University College London, London, NW1 2DA UK; 2 Department of Microbiology, Queen Elizabeth Hospital Birmingham, University Hospitals Birmingham NHS Foundation Trust, Mindelsohn Way, Birmingham, B15 2TH UK; 3 Health Informatics, University Hospitals Birmingham NHS Foundation Trust, 11-13 Frederick Road, Edgbaston, Birmingham, B15 1JD UK; 4 Institute of Clinical Trials and Methodology, University College London, 90 High Holborn, London, WC1V 6LJ UK; 5 Institute of Epidemiology & Healthcare, University College London, 1-19 Torrington Place, London, WC1E 7HB UK

**Keywords:** Antimicrobial resistance, Antimicrobial stewardship, Urinary tract infection, Emergency department, Electronic health records

## Abstract

**Background:**

Suspected urinary tract infection (UTI) syndromes are a common reason for empirical antibiotics to be prescribed in the Emergency Department (ED), but differentiating UTI from other conditions with a similar presentation is challenging. We investigated how often an ED diagnosis of UTI is confirmed clinically/microbiologically, and described conditions which present as UTI syndromes.

**Methods:**

Observational study using electronic health records from patients who attended the ED with suspected UTI and had a urine sample submitted for culture. We compared the ED diagnosis to diagnosis at discharge from hospital (ICD-10 codes), and estimated the proportion of cases with clinical/microbiological evidence of UTI.

**Results:**

Two hundred eighty nine patients had an ED diagnosis of UTI syndrome comprising: lower UTI (191), pyelonephritis (56) and urosepsis (42). In patients admitted to hospital with an ED diagnosis of lower UTI, pyelonephritis or urosepsis, clinical/microbiological evidence of UTI was lacking in 61/103, 33/54 and 31/42 cases respectively. The ED diagnosis was concordant with the main reason for admission in less than 40% of patients with UTI syndromes, and antibiotics were stopped within 72 h in 37/161 patients.

**Conclusions:**

Clinical/microbiological evidence of UTI was lacking in 60–70% of patients, suggesting scope to revise empirical prescribing decisions for UTI syndromes in light of microbial culture and clinical progression.

## Background

Urinary tract infection syndromes are a major cause of ED attendance and hospital admission [1]. Concerns over delaying antibiotic treatment for severe infection in patients with suspected UTI means that clinicians have a low threshold for initiating antibiotics in the ED, however differentiating UTI from other conditions with a similar presentation can be challenging. These difficulties are compounded by conflicting messages which promote rapid instigation of antibiotics for suspected sepsis on the one hand, and prudent antibiotic use on the other. Diagnosing UTI is particularly challenging because patients may present with atypical signs and symptoms [2], diagnostic tests based on microbial culture of urine or blood take 48–72 h, the finding of bacteriuria without associated urinary symptoms does not usually warrant antibiotic treatment [3], and rapid urine dipstick tests have a poor positive predictive value for bacteriuria [4].

Previous studies have suggested that UTIs may be both over and under-diagnosed in the ED [5, 6], particularly in the elderly [7, 8], with one study suggesting that > 40% of patients aged > 65 years who had been diagnosed with UTI had no evidence of this condition [9]. Although a non-infectious cause is established for many of these cases, antibiotics are often continued unnecessarily, which drives the emergence of anti-bacterial resistance (ABR).

Using electronic health records supplemented by medical note review, we set out to estimate the frequency of over-diagnosis of UTI syndromes in the ED, and to describe the conditions which present as UTI in the ED in order to estimate the potential to reduce antibiotic prescribing by stopping antibiotics early in patients with no evidence of bacterial infection.

## Methods

### Study design and source of data

We undertook a retrospective cohort study in patients who were investigated for suspected UTI syndromes between 1st January 2014 and 30th June 2017 at the Emergency Department (ED) of the Queen Elizabeth Hospital Birmingham (QEHB), which is part of University Hospitals Birmingham NHS Foundation Trust. QEHB is the largest single site hospital in England with over 1200 inpatient beds and a local resident population of around 1.3 million people, which is ethnically diverse with high levels of social deprivation. Individuals were eligible for inclusion in the study if they attended the ED and had a urine sample submitted for microbiological culture from the ED (defined as receipt of a urine culture in the laboratory ±2 days from ED attendance since there can be a delay between sample collection and when the urine sample is recorded in the laboratory system). From this group we used ED diagnostic codes to identify patients with suspected UTI, and used simple random sampling to select 300 patients who were discharged from the ED and 700 patients who had been admitted to hospital. ED diagnostic codes represent the main reason for the patients ED attendance, are recorded by the ED physician and distinguish between urological conditions and different UTI syndromes.

### Patients with suspected UTI who were discharged from the ED

We estimated the proportion of patients discharged from the ED with suspected UTI who had clinical/microbiological evidence of this diagnosis, based on the presence of urinary symptoms and microbial culture of urine and/or blood. A single investigator (LS) evaluated whether urinary symptoms had been recorded in the ED notes, considering: dysuria, frequency, hesitancy, urinary retention, difficulty passing urine, haematuria and malodorous urine. Information was also extracted about pain localised to the back, abdomen, flank, loin and suprapubic region. Symptoms were classified as probable if patients reported at least two urinary symptoms or a urinary symptom and pain, and possible if patients reported only one urinary symptom or only pain. Patients with no record of relevant pain or urinary symptoms were classified as having no symptoms of UTI. This information was integrated with results of urinary white cell count and microbial culture of urine and/or blood to categorise patients as probable, possible or no evidence of UTI (Table 1).

**Table 1:** Definition of clinical diagnosis of urinary tract infection syndromes

**Probable UTI**	1. Probable or possible symptoms^a^ of UTI and either: a. Urinary WCC > threshold^b^ and urinary culture > 10^3^cfu/mL; or b. Urinary culture > 10^5^ cfu/mL (irrespective of urinary WCC result) 2. Urinary pathogen identified from both blood and urine samples *Pregnant women only:* 1.a. or 1.b. in the absence of urinary symptoms^a^
**Possible UTI**	1. No urinary symptoms^a^ and either: a. Urinary WCC > threshold^b^ and urinary culture > 10^3^cfu/mL; or b. Urinary culture > 10^5^ cfu/mL 2. Probable or possible urinary symptoms^a^ and urinary culture >10^3^cfu/mL and urinary WCC not performed.
**No evidence of UTI**	1. Urine culture < 10^3^ 2. Urine culture < 10^5^ and urinary WCC < threshold^b^ 3. No urinary symptoms^a^, urine culture < 10^5^ and urinary WCC not performed

^a^ Urinary symptoms include: dysuria, urinary frequency, urgency, hesitancy, urinary retention, difficulty passing urine, haematuria, and malodorous urine. We further considered the following relevant pain symptoms reported by the patients: abdominal, back, flank, loin, and suprapubic. Symptoms were classified as probable if patients reported at least two urinary symptoms or a urinary symptom and pain. Symptoms were classified as possible if patients reported only one urinary symptom or only pain

^b^ In October 2015 the microbiology laboratory revised their standard operating procedure for urine culture which included adjusting the urinary white cell count threshold for undertaking urine culture from 40 cells/μL to 80 cells/μL

### Patients admitted to hospital for suspected UTI

For patients admitted to hospital with suspected UTI, we compared two approaches to estimate scope for improving antibiotic prescribing for UTI. First we estimated the proportion of patients with microbiological/clinical evidence of UTI as previously described (Table 1). Next we compared diagnoses made in the ED to the primary ICD-10 diagnostic code, which can identify patients who were admitted for treatment of a UTI syndrome, using code lists derived from the NHS outcomes framework [10]. The primary ICD-10 code represents the main reason for the patient’s admission and is entered retrospectively after the patient has left hospital based on information recorded in the medical record. Consequently the primary ICD-10 code and the ED diagnosis may be discordant when test results and the patient’s clinical progression in hospital make it clear that the ED diagnosis was incorrect.

Investigations, test results and antibiotic prescriptions that were ordered in the ED were not recorded electronically, consequently information on urinary symptoms, results of urinalysis, antibiotic prescriptions, presence of an indwelling urinary catheter, Standardised Early Warning Score (SEWS) [11], and temperature were transcribed from the medical notes and entered into a database which was stored securely at QEHB. Demographic data (age, gender, index of multiple deprivation-IMD), were obtained from the electronic health record in addition to microbiological culture and sensitivity results from urine and blood. Antibiotics were categorised as broad (carbapenems, cephalosporins, co-amoxiclav, colistin, piperacillin/tazobactam) or narrow spectrum (all other antibiotics) [12]. We further obtained all ICD-10 and OPCS codes for the 5-year period preceding admission in order to calculate Charlson Comorbidity Indices. Patients with no relevant code recorded in the 5 years prior to admission were classified as not having any comorbidities.

Microbiological culture of urine and/or blood was undertaken in accordance with standard clinical laboratory procedures current at the time (UK Standards for Microbiology Investigations: SMI B41, Investigation of Urine; SMI B37: investigation of blood cultures (for organisms other than *Mycobacterium* species) [13]. Urine cell counts were done on the UF100 Urine Analysis (Sysmex, Milton Keynes, UK) until September 2015 and subsequently on its newer version UF1000i. Cell counts were not possible for urine samples less than 4 mL or for samples too viscous to pass through the instrument. Samples for which cell counts could not be done were always cultured. When cell counts were available, only urines with white blood cell counts above a threshold value were cultured. At the start of the study the threshold value was 40/μL **or** bacteria counts > 4000/μL. This was adjusted to 80/μL **or** bacteria counts > 8000/μL following the introduction of a revised standard operating procedure in the microbiology laboratory in October 2015. Semiquantitative culture was done on Chromid® CPS® Elite agar (bioMérieux, Basingstoke, UK). Microbial samples of urine or blood were classified as multidrug-resistant based on criteria agreed by international experts in a joint initiative of the European Centre for Disease Prevention and Control, and the Centers for Disease Control and Prevention [14].

### Analytical approach

The primary outcome was the proportion of patients with an ED diagnosis of suspected UTI who met clinical/microbiological criteria for possible or probable UTI (Table 1), comparing patients who were and were not admitted to hospital. Secondary outcomes in patients admitted to hospital with an ED diagnosis of suspected UTI included: the proportion without a primary ICD-10 code for a UTI syndrome; the proportion with a primary ICD-10 code for a non-infectious condition; and the proportion stopping antibiotics within 72 h following admission (defined as the first calendar day with no record of antibiotic treatment). We undertook sensitivity analysis by extending comparison of the ED diagnosis and ICD-10 code to include the highest ranked secondary diagnostic code, since conditions such as urosepsis may be recorded using two codes.

For patients who had been admitted to hospital, we generated alluvial plots to illustrate the relationship between diagnoses that were made in the ED, and the patients’ main reason for admission to hospital based on ICD-10 coding, comparing patients with and without microbiological evidence of UTI.

## Results

**Fig. 1:**
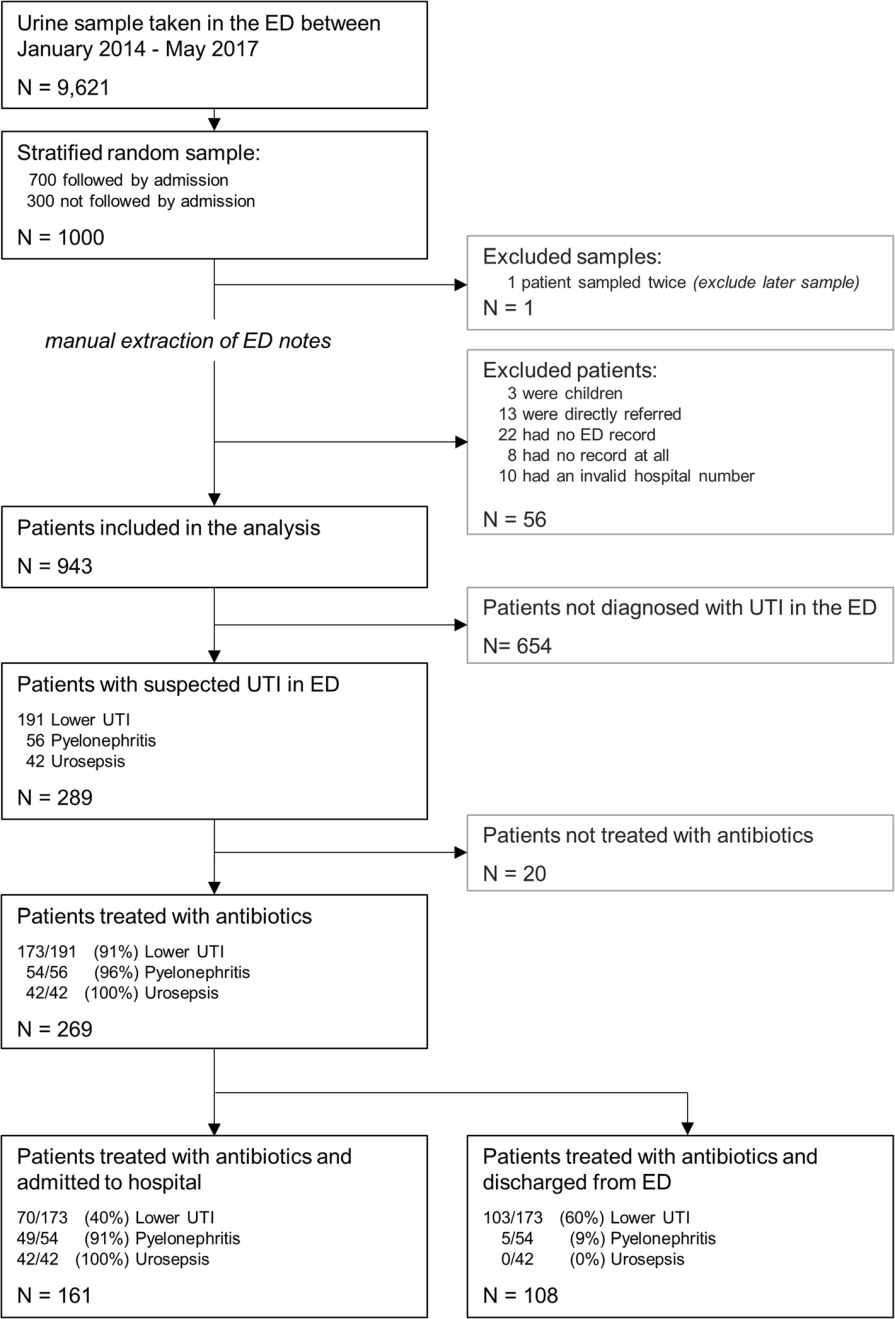
Study flow chart

Nine hundred forty three patients met the study inclusion criteria and 289 patients were diagnosed with a UTI syndrome in the ED (Fig. 1). Patients who were admitted to hospital were older compared to those discharged from the ED (mean age 72 versus 44 years), more likely to be male (39.8% versus 29.8%) and to have at least one comorbidity (44.9% versus 24.3%), (Table 2). Patients who were discharged from the ED were also more likely to have a positive urinalysis compared to patients who were admitted to hospital (72.1% versus 66.4%). Overall there were 191 patients with an ED diagnosis of lower UTI, 56 cases with pyelonephritis and 42 patients with urosepsis (Table 3).

**Table 2:** Baseline characteristics of the study population, stratified by admission status

	Not admitted to hospital (discharged directly from ED)	Admitted to hospital
**Total**	272 (100.0)	671 (100.0)
**Age**^a^	44.0 (26.0–71.0)	72.00 (50.0–84.0)
**Female**	191 (70.2)	404 (60.2)
**Social deprivation**		
Least deprived (Q1-Q3)	16 (21.6)	221 (33.1)
Most deprived (Q4-Q5)	58 (78.4)	446 (66.9)
Missing	198	4
**Ethnicity**		
White	164 (65.9)	532 (82.0)
Asian	47 (18.9)	74 (11.4)
Other	38 (15.3)	43 (6.6)
Missing	23	22
**CCI**		
0	206 (75.7)	370 (55.1)
1–2	34 (12.5)	170 (25.3)
> 2	32 (11.8)	131 (19.5)
**Urinary catheter in situ**	21 (7.7)	53 (7.9)
**Pregnant**	33 (12.1)	7 (1.0)
**SEWS**^a^	0.00 (0.0–1.0)	1.00 (0.0–2.0)
Missing	95	10
**Temperature**^a^	36.50 (36.1–36.9)	36.50 (36.0–37.2)
Missing	12	3
**Positive urinalysis**^b^	196 (72.1)	446 (66.5)
**Symptoms**		
Frequency	30 (11.0)	68 (10.1)
Dysuria	63 (23.2)	88 (13.1)
Hesitancy	2 (0.7)	1 (0.1)
Malodorous urine	1 (0.4)	16 (2.4)
Urinary retention	2 (0.7)	5 (0.7)
Difficulty passing urine	9 (3.3)	20 (3.0)
Pain in back or abdomen	79 (29.0)	144 (21.5)

^a^ Numerical variables were summarized using median (Interquartile range – IQR); ^b^ Defined as the presence of leukocytes and/or nitrates on urinalysis

**Table 3:** Antibiotic use and microbiological outcomes in patients with an ED diagnosis of UTI syndromes

	UTI		Pyelonephritis	Urosepsis
	Admitted	Not admitted		
**Total**	83 (100.0)	108 (100.0)	56 (100.0)	42 (100.0)
**Empirical antibiotic**^a^				
Piperacillin / tazobactam	25 (33.3)	1 (1.0)	42 (72.4)	30 (62.5)
Nitrofurantoin	15 (20.0)	35 (33.7)	0 (0.0)	1 (2.1)
Ciprofloxacin	10 (13.3)	18 (17.3)	11 (19.0)	4 (8.3)
Trimethoprim	6 (8.0)	19 (18.3)	0 (0.0)	0 (0.0)
Amoxicillin / clavulanic acid	7 (9.3)	9 (8.7)	3 (5.2)	3 (6.2)
Other	12 (16.0)	22 (21.2)	2 (3.4)	10 (20.8)
**Urinary culture results**^a^				
Positive (> 10^5^ cfu/mL)	22 (26.5)	43 (39.8)	20 (35.7)	10 (23.8)
* E. coli*	18 (21.7)	34 (31.5)	14 (25.0)	9 (21.4)
*K. pneumoniae*	0 (0.0)	1 (0.9)	2 (3.6)	0 (0.0)
*P. mirabilis*	1 (1.2)	2 (1.9)	1 (1.8)	0 (0.0)
Other	3 (3.6)	6 (5.6)	3 (5.4)	1 (2.4)
Heavy mixed growth / no growth	33 (39.8)	35 (32.4)	24 (42.9)	19 (45.2)
Culture not performed	28 (33.7)	30 (27.8)	12 (21.4)	13 (31.0)
Resistant urine pathogens^c^	8 (36.4)	11 (25.6)	3 (15.0)	5 (50.0)
**Urinary white cell count**				
< threshold^b^	24 (32.9)	27 (27.3)	12 (23.1)	14 (35.9)
> threshold^b^	49 (67.1)	72 (72.7)	40 (76.9)	25 (64.1)
Not performed	10	9	4	3
**Blood culture results**^a^				
Positive	4 (4.8)	1 (0.9)	2 (3.6)	7 (16.7)
*E. coli*	0 (0.0)	0 (0.0)	1 (1.8)	3 (7.1)
Other	4 (4.8)	1 (0.9)	1 (1.8)	4 (9.5)
No growth	19 (22.9)	4 (3.7)	28 (50.0)	28 (66.7)
Culture not taken	60 (72.3)	103 (95.4)	26 (46.4)	7 (16.7)
Resistant blood pathogens	0 (0.0)	0 (0.0)	0 (0.0)	2 (28.6)
**Antibiotics stopped**				
At admission	17 (24.3)	–	4 (8.2)	4 (9.5)
< 72 h	24 (34.3)	–	5 (10.2)	8 (19.0)

^a^ Multiple antibiotics may be given to a single patient and multiple specimen might be identified from a single patient’s specimen

^b^ Threshold for undertaking urine culture was adjusted from 40 urinary white cells/μL to 80 urinary white cells/μL in October 2015, following the introduction of a new laboratory standardised operating procedure.

^c^ Heavy mixed growth: samples in which there were multiple organisms of >1 species, where no single species predominates and colonies are so numerous as to be unquantifiable by the culture method used. These samples are most likely to represent contamination

### Comparison of the ED diagnosis to clinical/microbiological criteria

There were 191 patients with an ED diagnosis of lower UTI, 56 cases with pyelonephritis and 42 patients with urosepsis (Table 3). Approximately one half (83/176) of the patients who were admitted to hospital, and nearly all (108/113; 95.6%) patients who were discharged from the ED were diagnosed with lower UTI. For patients thought to have lower UTI and who were started on antibiotic treatment in the ED, the ED diagnosis of lower UTI was not supported by clinical/microbiological evidence in 49/70 (70.0%) of admitted patients and 61/103 (59.2%) non-admitted patients.

Similarly, 33/54 (61.1%) of patients with ED diagnosed pyelonephritis and 31/42 (73.8%) patients with ED diagnosed urosepsis lacked clinical/microbiological evidence of this infection.

In patients who were admitted to hospital with ED diagnosed lower UTI and treated with empirical antibiotics, antibiotics were stopped at or within 72 h following admission in 17/70 (24.3%) and 24/70 (34.3%) respectively. Antibiotics were stopped within 72 h in 5/49 (10.2%) of patients with ED diagnosed pyelonephritis and 8/42 (19.0%) of patients with ED diagnosed urosepsis.

### Comparison of ED diagnoses and ICD-10 codes

For patients who were admitted to hospital we compared the ED diagnosis to the main reason for the patient’s admission based on ICD-10 codes. In this group, lower UTI remained the main reason for admission in 33/83 cases (39.8%) who were diagnosed with this condition in the ED (Additional file 1, Fig. 2) and 3 patients in this group were diagnosed with pyelonephritis or urosepsis. However, the main reason for admission was non-infectious in 40.9% (95% CI: 30.3–52.3%) of patients with an ED diagnosis of lower UTI.

**Fig. 2:**
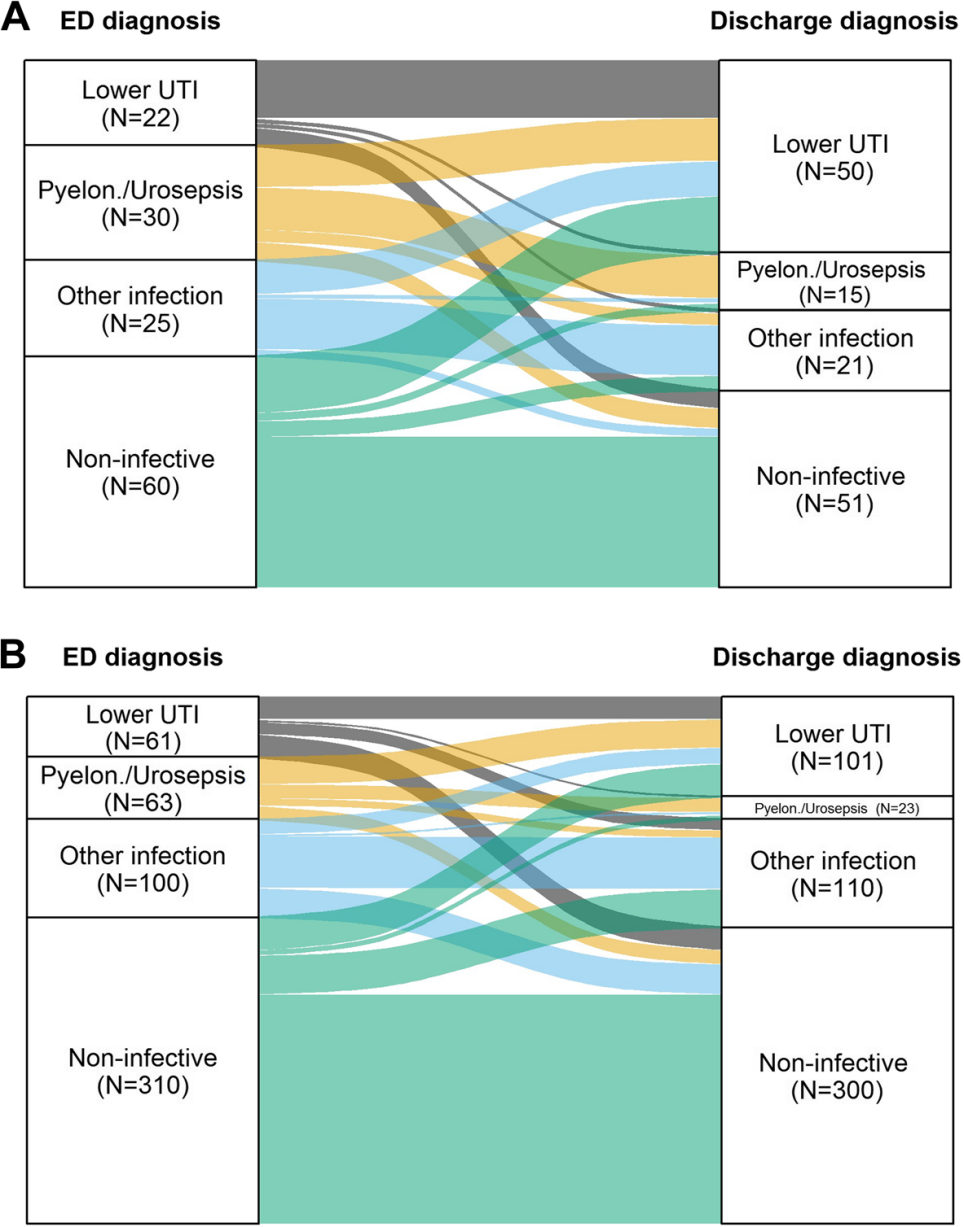
Comparison of the diagnosis that was made in the Emergency Department and the reason for the patient’s admission to hospital (based on ICD-10 code), in **a**) patients with clinical/microbiological evidence of UTI and **b**) patients without clinical/microbiological evidence of UTI. This figure illustrates how diagnoses that are made in the ED are revised during admission in light of test results and clinical progression. For example, in Fig. 2a 30 patients with clinical/ microbiological evidence of UTI are diagnosed with pyelonephritis in the ED. The diagnosis of pyelonephritis is “correct” in 11 (36.7%) cases, but it is revised to lower UTI or a non-infectious condition in 11 (36.7%) and 5 (16.7%) cases respectively. In Fig. 2b, 61 cases are diagnosed with lower UTI in the ED (but none of these individuals have clinical/microbiological evidence of UTI). The diagnosis of lower UTI is confirmed 23 (37.7%) cases, but 12 patients (19.7%) are diagnosed with a different infection and 24 (39.3%) are diagnosed with a non-infectious condition

Pyelonephritis remained the main reason for admission in about a quarter (13/51) patients who were diagnosed with this condition in the ED. The diagnosis was downgraded to lower UTI in 20 (39.2, 95% CI: 25.8–53.9%) patients with this ED diagnosis and one patient was diagnosed with urosepsis. The main reason for admission was non-infectious in 14 patients (27.5, 95% CI: 15.9–41.7%).

Forty two patients were diagnosed with urosepsis in the ED, but this remained the main reason for admission in just 2 patients, with 3 cases of pyelonephritis. The diagnosis was downgraded to lower UTI in almost half of patients (20/42) who had been diagnosed with urosepsis in the ED and the main reason for admission was non-infectious in 11 cases (26.2, 95% CI:13.9–42.0%).

Sensitivity analysis comparing the ED diagnosis against both the primary and secondary ICD-10 diagnostic code slightly reduced the proportion of patients who had a non-infectious reason for admission to 29/83 (34.9, 95% CI: 24.8–46.2%) of patients with an ED diagnosis of lower UTI, 12/51 (23.5, 95% CI: 12.8–37.5%) with an ED diagnosis of pyelonephritis, and 7/42 (16.7, 95% CI: 7.0–31.3%) with an ED diagnosis of urosepsis. The relationship between the diagnosis of UTI in the ED and the main reason for admission based on ICD-10 coding is illustrated in Fig. 2a and b.

## Discussion

Clinical/microbiological evidence of infection was lacking in > 60% of patients who were treated empirically for UTI syndromes in the ED, but antibiotics were stopped within 72 h in less than one-quarter of patients. Our findings suggest there is scope to further reduce antibiotic prescribing for this common condition, and highlights the potential impact of reviewing and revising antibiotic prescribing decisions in light of microbial culture and clinical progression.

There are many reasons why it is challenging to estimate how often UTI is over-diagnosed in the ED. Firstly there is a lack of consensus on diagnostic criteria for UTI which is compounded by the fact that around 20% of patients with genuine lower UTI will have negative urine cultures [15, 16]. Prior antibiotic treatment also reduces the likelihood of obtaining a microbial culture from urine or blood, and this may partly explain why we failed to identify a urinary pathogen in over half of patients with suspected UTI in this study. Using the medical notes we estimated that 9.4% (89/943) patients had received antibiotics prior to their ED attendance, but this is likely to be an underestimate as this information is poorly recorded. Previous studies in patients aged > 65 years have highlighted the discrepancy between an ED diagnosis of bacterial infection (including UTI) against standardized criteria for infection [7], and this is supported by a number of studies which have estimated the prevalence of “inappropriate” antibiotic prescribing in the ED to range from 30 to 40% [17, 18]. In common with these studies, we found that the reason for admission was not an infection in around 40% of patients with an ED diagnosis of lower UTI, suggesting that these patients are unlikely to have benefitted from antibiotic treatment. We also note that a proportion of cases diagnosed as “non-infectious” in the ED were subsequently allocated a primary ICD-10 code for UTI, suggesting that under-diagnosis of this condition also occurs. Our findings highlight the uncertainty around the diagnosis and diagnostic coding of UTI syndromes in secondary care, a problem which severely undermines the ability to implement and evaluate antimicrobial stewardship initiatives at scale.

We demonstrate a clear discrepancy between diagnoses that are made in the ED and clinical and microbiological information that accumulates during admission to hospital, which emphasises the importance of antibiotic prescribing review as a core element of hospital antibiotic stewardship programmes [19, 20]. Our findings also demonstrate the limitations of rapid diagnostic tests such as urinalysis that are widely used in the ED, since more than 60% of patients who were not diagnosed with a UTI syndrome had evidence of UTI on urinalysis i.e. detection of leukocytes and/or nitrites. Overall, antibiotics were stopped in less than one quarter of patients who were diagnosed with a UTI syndromes in the ED, despite the fact that an estimated 40% of these patients may not have benefitted from antibiotic therapy. The ED diagnosis of pyelonephritis and/or urosepsis was downgraded to UTI in 40–50% of patients once they had been admitted to hospital, highlighting the opportunity to de-escalate antibiotics. We were unable to estimate how often de-escalation took place in this study, due to the small number of patients with each type of UTI syndrome. However, recent national audits in patients with sepsis in England suggests that > 80% of antibiotic prescriptions are continued following antibiotic prescribing review at 48–72 h [12].

### Strengths and limitations

Diagnostic criteria for UTI syndromes are notoriously difficult with laboratories, and therefore different studies each employing different protocols. As in most UK laboratories, our laboratory used the UK Standards in Microbiology Investigations (SMI) for urine current at the time [13]. In its guidance for interpreting urine cultures, this SMI reflects the variation in interpretation that might be placed on urines that grow micro-organisms. In a range of growth from 10^2^ to 10^5^ cfu/mL, the nature of the specimen, the urine white cell count and the patient’s symptoms all need to be considered in assigning the likelihood of a urinary tract infection. Even the SMI states that interpretation may need to be supplemented with local reporting policies. It is possible that the laboratory algorithm that is used to determine whether samples undergo microbial culture may have underestimated the prevalence of bacteriuria in patients included in this study. Our approach may have erroneously classified patients with asymptomatic bacteriuria as having genuine UTI or have failed to identify patients with urinary symptoms where this information was not clearly recorded. In addition it is possible that some patients with an ED diagnosis of UTI did have this condition, but the diagnosis was superseded by a different complaint which was recorded as the primary ICD-10 code. We attempted to investigate this issue by undertaking a sensitivity analysis looking at secondary ICD-10 codes. We restricted our analysis to patients who had a urine sample submitted from the ED and acknowledge that only a proportion of patients with suspected UTI have a urine sample submitted for microbial culture. Preliminary, unpublished estimates from a related, ongoing research study at this site suggest a urine sample is submitted for culture from 44% of patients who are diagnosed with suspected UTI in the ED. It seems likely that there are reasons why doctors submit urine samples for culture in some patients but not others, potentially relating to diagnostic uncertainty, so it is unclear whether our findings can be generalised to all patients with suspected UTI. A further limitation of our analysis is that a number of conditions that are included in the Charlson Comorbidity Index are unlikely to be recorded in secondary care.

A strength of our approach is that fact that ED physicians use structured codes to record the diagnosis at discharge from the ED, increasing the likelihood that this information accurately represents the doctors’ decision making. We capitalised on the availability of high quality electronic health records at this site and also undertook a detailed review of the medical notes to extract information on urinary symptoms and antibiotics that were prescribed in the ED. However, a limitation of our approach is that this study was conducted at a site which was selected because of its electronic health records. Whilst there is a widely held view that clinical infection syndromes, and UTI’s in particular, are likely to be over-diagnosed in the ED, more sites would be required in order to generalise these findings beyond this hospital.

### Clinical implications and future research

Efforts to monitor and improve prescribing for UTI syndromes are undermined by the complexity of the diagnosis, and the difficulty in differentiating UTI from other conditions with a similar presentation. For these reasons, we emphasise the importance of reviewing antibiotic prescribing decisions in patients with suspected UTI, and the potential impact on total prescribing that could be achieved if this approach was adopted more widely. However, the diagnostic challenge of UTI could be addressed most effectively through the development of rapid diagnostics with sufficient negative predictive power to reliably rule out bacterial infection.

## Supplementary information

**Additional file 1.** Most common ICD-10 discharge diagnoses in patients who were admitted to hospital with an ED diagnosis of UTI syndrome.

## Data Availability

The data that support the findings of this study are available from University Hospitals Birmingham NHS Foundation Trust, but restrictions apply to the availability of these data, which are not publicly available. Data are however available from the authors upon reasonable request and with permission of University Hospitals Birmingham NHS Foundation Trust. Codelists and analytical code are available from the authors.

## Abbreviations

UTI: Urinary Tract Infection
ED: Emergency Department
ABR: Anti-bacterial resistance
QEHB: Queen Elizabeth Hospital Birmingham
ICD: International Classification of Diseases
Cfu/mL: Colony forming units per mL
WCC: White cell count
SEWS: Standardised early warning score
IMD: Index of Multiple Deprivation
OPCS: Classification of Interventions and Procedures
SMI: Standards for Microbiology Investigation
CCI: Charlson Comorbidity Index

## References

[1] Foxman B (2010). The epidemiology of urinary tract infection. Nat Rev Urol.

[2] Wilson ML, Gaido L (2004). Laboratory diagnosis of urinary tract infections in adult patients. Clin Infect Dis.

[3] Nicolle LE, Bradley S, Colgan R, Rice JC, Schaeffer A, Hooton TM (2005). Infectious Diseases Society of America guidelines for the diagnosis and treatment of asymptomatic bacteriuria in adults. Clin Infect Dis.

[4] Semeniuk H, Church D (1999). Evaluation of the leukocyte esterase and nitrite urine dipstick screening tests for detection of bacteriuria in women with suspected uncomplicated urinary tract infections. J Clin Microbiol.

[5] Kiyatkin D, Bessman E, McKenzie R (2016). Impact of antibiotic choices made in the emergency department on appropriateness of antibiotic treatment of urinary tract infections in hospitalized patients. J Hosp Med.

[6] Tomas ME, Getman D, Donskey CJ, Hecker MT (2015). Overdiagnosis of urinary tract infection and Underdiagnosis of sexually transmitted infection in adult women presenting to an emergency department. J Clin Microbiol.

[7] Caterino JM, Leininger R, Kline DM, Southerland LT, Khaliqdina S, Baugh CW (2017). Accuracy of current diagnostic criteria for acute bacterial infection in older adults in the emergency department. J Am Geriatr Soc.

[8] Caterino JM, Stevenson KB (2012). Disagreement between emergency physician and inpatient physician diagnosis of infection in older adults admitted from the emergency department. Acad Emerg Med.

[9] Woodford HJ, George J (2009). Diagnosis and management of urinary tract infection in hospitalized older people. J Am Geriatr Soc.

[10] NHS Digital. 3a Emergency admissions for acute conditions that should not usually require hospital admission - NHS Digital. https://digital.nhs.uk/data-and-information/publications/clinical-indicators/nhs-outcomes-framework/current/domain-3-helping-people-to-recover-from-episodes-of-ill-health-or-following-injury-nof/3a-emergency-admissions-for-acute-conditions-that-should-not-usually-require-hospital-admission. Accessed 29 Jul 2019.

[11] Paterson R, MacLeod DC, Thetford D, Beattie A, Graham C, Lam S (2006). Prediction of in-hospital mortality and length of stay using an early warning scoring system: clinical audit. Clin Med.

[12] Public Health England. English surveillance programme for antimicrobial utilisation and resistance (ESPAUR). https://improvement.nhs.uk/resources/english-surveillance-programme-antimicrobial-utilisation-and-resistance-espaur/. Accessed 29 Jul 2019.

[13] Public Health England (2014). Standards for microbiology investigations (UK SMI). GOV.UK.

[14] Magiorakos A-P, Srinivasan A, Carey RB, Carmeli Y, Falagas ME, Giske CG (2012). Multidrug-resistant, extensively drug-resistant and pandrug-resistant bacteria: an international expert proposal for interim standard definitions for acquired resistance. Clin Microbiol Infect.

[15] Ferry SA, Holm SE, Stenlund H, Lundholm R, Monsen TJ (2004). The natural course of uncomplicated lower urinary tract infection in women illustrated by a randomized placebo controlled study. Scand J Infect Dis.

[16] Kwon JH, Fausone MK, Du H, Robicsek A, Peterson LR (2012). Impact of laboratory-reported urine culture colony counts on the diagnosis and treatment of urinary tract infection for hospitalized patients. Am J Clin Pathol.

[17] Fleming-Dutra KE, Hersh AL, Shapiro DJ, Bartoces M, Enns EA, File TM (2016). Prevalence of inappropriate antibiotic prescriptions among US ambulatory care visits, 2010-2011. JAMA..

[18] Denny KJ, Gartside JG, Alcorn K, Cross JW, Maloney S, Keijzers G (2019). Appropriateness of antibiotic prescribing in the emergency department. J Antimicrob Chemother.

[19] US Centers for Disease Control. Core Elements of Hospital Antibiotic Stewardship Programs. https://www.cdc.gov/antibiotic-use/core-elements/hospital.html. Accessed 29 Jul 2019.

[20] National Institute of Health and Care Excellence. Antimicrobial stewardship: systems and processes for effective antimicrobial medicine use. https://www.nice.org.uk/guidance/NG15/chapter/1-Recommendations#all-antimicrobials. Accessed 29 Jul 2019.

