# Supplementary material for: Diagnostic uncertainty and urinary tract infection in the emergency department: a cohort study from a UK hospital
Source: BMC Emerg Med. 2020 May 19;20:40. doi: 10.1186/s12873-020-00333-y (PMC7238572; doi:10.1186/s12873-020-00333-y)
Supplement: Supplementary file 1 — Additional file 1. Most common ICD-10 discharge diagnoses in patients who were admitted to hospital with an ED diagnosis of UTI syndrome. [file 12873_2020_333_MOESM1_ESM.docx]

**Additional File 1 – Most common ICD-10 discharge diagnoses in patients who were admitted to hospital with an ED diagnosis of UTI syndrome**

|  |  |  |
| --- | --- | --- |
| **Primary ICD-10 diagnosis** | **N** | **%** |
|  |  |  |
|  |  |  |
| **ED: Lower UTI (n = 83*)**  Lower UTI | 33 | 39.8 |
| Other non-infectious diagnosis | 31 | 37.3 |
| Other infection | 13 | 15.7 |
| Other urological condition | 3 | 3.6 |
| Pyelonephritis | 2 | 2.4 |
| Urosepsis | 1 | 1.2 |
| **ED: Pyelonephritis (n = 51*)**  Lower UTI | 20 | 39.2 |
| Pyelonephritis | 13 | 25.5 |
| Other urological condition | 9 | 17.6 |
| Other non-infectious diagnosis | 5 | 9.8 |
| Other infection | 3 | 5.9 |
| Urosepsis | 1 | 2.0 |
| **ED: Urosepsis (n = 42*)**  Lower UTI | 20 | 47.6 |
| Other non-infectious diagnosis | 11 | 26.2 |
| Other infection | 6 | 14.3 |
| Pyelonephritis | 3 | 7.1 |
| Urosepsis | 2 | 4.8 |
|  |  |  |

* The denominator is the number of patients diagnosed with the syndrome in the ED and who were subsequently admitted to hospital, irrespective of whether antibiotic treatment was initiated in the ED.
